# Photoelectrochemical Behavior of Electrophoretically Deposited Hematite Thin Films Modified with Ti(IV)

**DOI:** 10.3390/molecules21070942

**Published:** 2016-07-20

**Authors:** Nicola Dalle Carbonare, Rita Boaretto, Stefano Caramori, Roberto Argazzi, Maurizio Dal Colle, Luca Pasquini, Renzo Bertoncello, Marcello Marelli, Claudio Evangelisti, Carlo Alberto Bignozzi

**Affiliations:** 1 Department of Chemical and Pharmaceutical Sciences, University of Ferrara, Via Fossato di Mortara 11–17, 44121 Ferrara, Italy; bar@unife.it (R.B.); mau@unife.it (M.D.C.); g4s@unife.it (C.A.B.); 2 CNR/ISOF c/o Department of Chemical and Pharmaceutical Sciences, University of Ferrara, Via Fossato di Mortara 11–17, 44121 Ferrara, Italy; agr@unife.it; 3 Department of Physics and Astronomy, University of Bologna, Viale Berti Pichat 6/2, 40127 Bologna, Italy; luca.pasquini@unibo.it; 4 Department of Chemical Sciences, University of Padua, Via F. Marzolo 1, 35131 Padua, Italy; renzo.bertoncello@unipd.it; 5 CNR ISTM, Via Fantoli 16/15, 20138 Milan, Italy; m.marelli@istm.cnr.it (M.M.); claudio.evangelisti@istm.cnr.it (C.E.)

**Keywords:** electrophoresis, hematite, titanium, doping, passivation, water splitting, EIS

## Abstract

Doping hematite with different elements is a common strategy to improve the electrocatalytic activity towards the water oxidation reaction, although the exact effect of these external agents is not yet clearly understood. Using a feasible electrophoretic procedure, we prepared modified hematite films by introducing in the deposition solution Ti(IV) butoxide. Photoelectrochemical performances of all the modified electrodes were superior to the unmodified one, with a 4-fold increase in the photocurrent at 0.65 V vs. SCE in 0.1 M NaOH (pH 13.3) for the 5% Ti-modified electrode, which was the best performing electrode. Subsequent functionalization with an iron-based catalyst led, at the same potential, to a photocurrent of ca. 1.5 mA·cm^−2^, one of the highest achieved with materials based on solution processing in the absence of precious elements. AFM, XPS, TEM and XANES analyses revealed the formation of different Ti(IV) oxide phases on the hematite surface, that can reduce surface state recombination and enhance hole injection through local surface field effects, as confirmed by electrochemical impedance analysis.

## 1. Introduction

Crystalline hematite (α-Fe_2_O_3_) is an Earth-abundant indirect n-type semiconductor displaying a band gap of 2.2 eV, useful for collecting a large fraction of visible photons, with frontier energy levels suitably aligned for carrying out the photoelectrochemical water oxidation reaction under basic conditions in which a very good material stability under oxygen evolving conditions is found [1]. Despite these features hematite has a number of inherent limitations which demand bulk and surface engineering for a successful exploitation of α-Fe_2_O_3_ in energy conversion processes. Indeed, as a Mott insulator, α-Fe_2_O_3_ suffers from slow carrier transport, an inadequate feature for photo- electrochemical (PEC) applications. Different strategies have been employed to enhance the efficiency of hematite towards the photoelectrochemical water oxidation, including nanostructuring, to generate structures having a size compatible with its short carrier diffusion length [2] and modifications of the semiconductor surface with co-catalysts [3,4,5] in order to improve the slow kinetics of water oxidation, reducing both the high charge transfer overpotential and minimizing the recombination via surface trap states [6,7]. Improved photoelectrochemical activity has been also observed by using ultrathin layers of different metal oxides deposited at the FTO/hematite interface [8,9,10]. Among the above listed strategies, additional efforts toward improving the charge collection efficiency consist of intentionally incorporating impurity atoms into the hematite lattice. There are numerous examples of enhanced water splitting activity upon introduction of high levels (0.5%–20% atomic) of impurities such as Si, Pt, Mn, Cr, Al, Ge, W, Rh and Ag [11,12,13,14,15,16,17,18,19]. Doping has been mostly achieved by sputtering [11], chemical deposition [20], pulsed laser deposition (PLD) [21] and hydrothermal methods [22], showing in all cases a significant photocurrent increase. 

Regarding titanium doping, there are several ways by which the dopant can improve, in principle, the performance of hematite films, although the specific cause of these improvements often remained elusive. In particular, the dopant could increase the electronic density and thus the conductivity of the pristine semiconductor, but also the minority carrier mobility and/or their lifetime, allowing more holes to reach the semiconductor-liquid junction (SCLJ). For example, Wang et al. [20] noticed that titanium acts as n-type dopant causing an increase of the electron concentration, while Hahn et al. [23] attributed the high efficiency of titanium-doped hematite films to both the improved electron transport within the bulk of the film and the suppression of the recombination at the SCLJ, because of a stronger electric field near the surface. Furthermore, Rioult et al. [24] observed an increase of the carrier concentration combined with higher carrier diffusion lengths in Ti-doped epitaxial hematite thin films. Dopants can also improve the crystallinity of the hematite lattice or act as structural directing agents to produce nanostructured architectures with increased light absorption capabilities. Miao et al. [25] reported that Ti doping induces a change in nanostructure size and shape and Deng et al. [26] found a modification of the type of hematite nanostructure and crystallographic orientation upon incorporation of titanium. However, it seems more likely that the overall effect should be ascribed to a combination of different effects, both on morphology and bulk properties, as suggested by Zandi et al. [27].

The dopants can also passivate surface states and/or grain boundaries, thus reducing surface state recombination. Franking et al. [28] used a titanium post-treatment through drop casting of a Ti(IV) precursor onto hematite electrodes, showing both an increased carrier density and surface state passivation. Actually, post-synthetic treatments followed by high temperature annealing, are more likely responsible for the formation of a TiO_2_ pure (titania) or mixed phase on the hematite surface, probably due to the hydrolysis and successive oxidation of the titanium precursor present on the hematite surface. Ahmed et al. [29] has recently reported enhancements in the photoactivity of hematite, after deposition of a thin TiO_2_ layer using a water-soluble titanium complex, ascribed to the suppression of surface electron-hole recombination. These overlayers should be thin enough (<5 nm) to allow a tunneling charge transfer from the semiconductor surface to the solution, due to the energetic mismatch between hematite and titania energy bands. In 2015, Morante et al. [30] prepared hematite/titania composite thin films with different molar ratios (from 0% to up 20%), claiming that both the improved conductivity and the presence of a photoactive pseudo-brookite (Fe_2_TiO_5_) phase on the hematite surface were responsible for the increased performances of doped samples, showing that the introduction of titanium can account for multiple effects having photoelectrochemical relevance. Titanium (IV) ions might behave both as substitutional dopants, resulting in the substitution of iron cations within the hematite lattice and as precursors of a pure or mixed oxide overlayer, facilitating the hole transfer through the SCLJ. In fact, the surface modification of semiconductor photoelectrodes with passivating overlayers is an effective strategy to favor the charge-separation and charge-transfer processes across the SCLJ. For instance, the stability and the efficiency for water oxidation of Si, GaAs and GaP photoanodes can be greatly enhanced by coating their surfaces with a TiO_2_ overlayer using atomic layer deposition (ALD) [31].

We thus decided to modify a reported electrophoretic deposition methodology [32] to investigate the effect of the incorporation of titanium in mesoporous hematite films, by using Ti(IV) butoxide as precursor. This solution-based procedure allows us to easily vary the amount of titanium added, obtaining different nominally modified samples. Besides being easy to implement and scale up, this solution procedure can also be flexibly employed to screen other metals in the form of soluble precursors. All Ti(IV)-modified hematite samples show an improved photoactivity with respect to the un-modified reference electrodes, reaching the best performance in the presence of a nominal 5% Ti/Fe ratio, exhibiting a 4-fold photocurrent enhancement with respect to the un-treated electrode. AFM, XPS, TEM and XANES analysis reveal the predominating presence of TiO_2_ in the form of rutile that covers the iron oxide nanoparticles. The photoelectrochemical investigation, carried out by electrochemical impedance spectroscopy (EIS), suggests that the improvement in the photoactivity can be directly ascribed to the formation of the interface based on titanium oxide and hematite, which favors hole injection from the semiconductor surface into the electrolyte through space charge and surface dipole effects. The further functionalization with an amorphous iron-based catalyst, Fe-OEC [5], produces modified electrodes able to reach ca. 1.5 mA·cm^−2^ at 0.65 V vs. SCE in NaOH 0.1 M (pH 13.3), one of the highest photocurrent achieved by employing only solution based procedure and without the addition of precious elements.

## 2. Results

### 2.1. AFM, XPS, TEM and XANES Characterization

**Figure 1:**
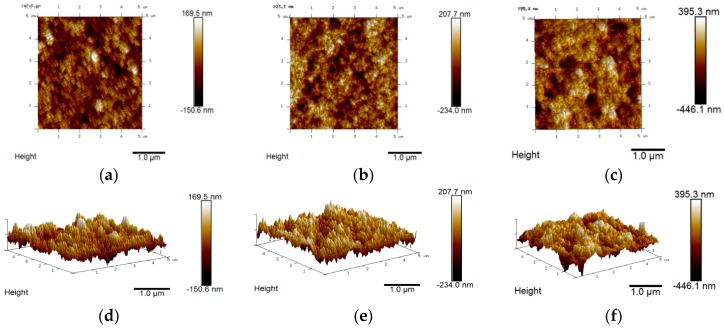
AFM top view images and 3D maps of (**a**,**d**) unmodified, (**b**,**e**) 5% and (**c**,**f**) 10%-Ti modified hematite electrodes.

Detailed mapping of the un-modified and the 5% and 10% Ti-modified hematite samples are presented in Figure 1. Morphological differences of the semiconductor surface are evident from both the top-view AFM images and the 3D maps taken for a 5 μm × 5 μm area. In the unmodified sample (Figure 1a,d), the surface is quite homogeneous, with iron oxide rods aligned perpendicularly to the basal plane with an approximate diameter <100 nm and clustered together in bigger domains. Although the hydrothermal growth of iron oxide nanoparticles does not allow to rigorously control the size of the nanoparticles, AFM images reveal that the rods are quite homogeneously distributed in both shape and size. When titanium is added, the nanoparticles appear more intimately sintered together, resulting in bigger lumps having a rounder and smoother shape. These features are more evident in the 10% Ti-modified sample (Figure 1c,f), where the surface roughness appears to be less evident due to the filling of the space between the nanoparticle domains. This may lead to a better charge transport across the fused nanostructures through the reduction of grain boundaries with respect to the unmodified sample (0% of titanium added).

**Figure 2:**
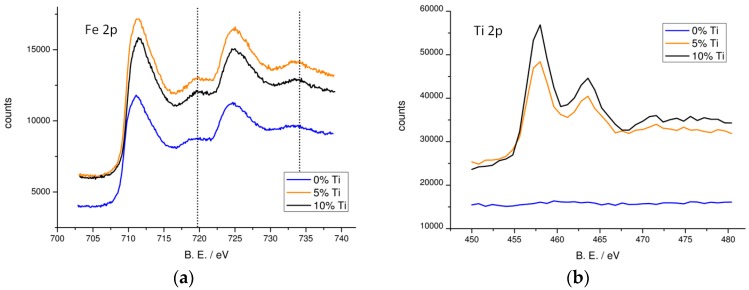
XPS spectra in the Fe and Ti energy region. (**a**) Fe 2p and (**b**) Ti 2p core level XPS spectra for un-modified (blue line), 5% (orange line) and 10% Ti-modified (black line) hematite samples. Dotted bars in (**a**) indicate Fe^3+^ shake-up peaks. Measurements have been performed with a 45° take off angle.

In order to analyze the surface of the material in search of changes upon Ti incorporation, both unmodified and Ti-modified (5% and 10%) hematite films have been investigated by XPS. Binding energies (B.E.) were calibrated on C1s peak (B.E. = 284.8 eV) (Figure S1 in the Supplementary Materials). The XPS spectra of the Fe 2p core are reported in Figure 2. They exhibit binding energies of 711.3 and 725.0 eV corresponding to the Fe 2p_3/2_ and Fe 2p_1/2_ lines, respectively. Typical Fe^3+^ shake-up satellites appear correctly separated by 8 eV from the main Fe 2p peaks (dotted bars in Figure 2a) [33], indicating that Fe^3+^ is the dominant ionic species in all the films. In the Ti(IV)-modified samples (orange and black lines) it is difficult to detect the presence of Fe^2+^ species which would result from incorporation of titanium in the hematite lattice (Fe^3+^ + Ti^4+^ → Fe^2+^ + Ti^3+^) [34] due to the range of diagnostic binding energies for Fe^2+^ species which substantially overlap with the much stronger Fe^3+^ peaks. Furthermore, the Fe 2p signals do not exhibit substantial peak shifts upon Ti incorporation, suggesting that Fe^3+^ cations experience a quite similar chemical neighborhood in both the modified and un-modified samples. Figure 2 also shows the Ti 2p core signal for the modified samples, split into the 2p_3/2_ (B.E. = 458.7 eV) and 2p_1/2_ (B.E. = 464.4 eV) peaks. No peaks are detected in this region for the un-modified hematite film (blue line in Figure 2b). The presence of the two Ti 2p components is consistent with the valence of Ti^4+^ in a titanium oxide phase [29], however the FWHM of these peaks (1.47 eV) is larger with respect to a pure TiO_2_ (rutile) film (1.32 eV) reflecting the presence of multiple slightly different chemical surroundings of the Ti(IV) oxide phase cast on the surface of hematite films (Figure S2). The relative intensity of Ti 2p peak, evaluated with respect to the Fe 2p signal, increases from 31% to 50% by moving from the 5% to the 10% Ti-modified samples, indicating a thicker titanium oxide surface layer for the 10% Ti-modified sample. The relative Ti/Fe intensity only slightly changes when analyzing the electrodes with a 20° take off angle suggesting a good surface homogeneity in the different samples: the Ti/Fe ratio observed at 20° and 45° is nearly constant in the 5% sample whereas it increases by about 15% when observing the 10% Ti-modified sample at the lower take off angle, suggesting, in this latter case, the formation of a thicker titanium oxide layer.

**Figure 3:**
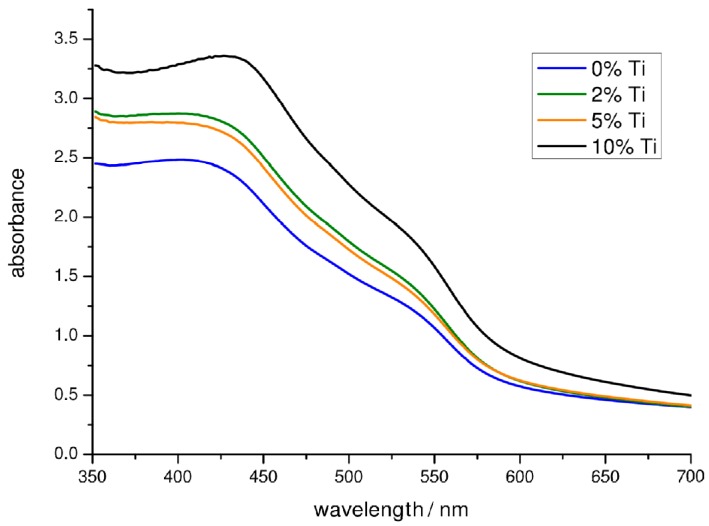
UV-Visible spectra of unmodified and Ti-modified hematite electrodes (2%, 5% and 10%).

The XPS analysis confirmed the presence of a titanium oxide phase onto the hematite surface, with a thickness that roughly depends on the initial amount of precursor used during the deposition step: the higher is the concentration of titanium(IV) butoxide the higher is the amount of titanium oxide detected. No evident proofs of the incorporation of titanium inside the hematite lattice are deducible at this stage. The presence of a thin layer of titanium oxide can be explained by the surface adsorption and hydrolysis of the titanium(IV) precursor during the electrophoretic deposition and the subsequent high temperature annealing. Furthermore, the presence of titanium does not seem to have any effect on the orientation of the iron oxide nanoparticles on the FTO. The presence of a thin titanium oxide shell on the top of the films is consistent with the smoother morphology observed from AFM images, helping to fill the gaps between the nanoparticle domains and forming larger conglomerates with improved adhesion and sintering of the nanoparticles, consistent with the spectrophotometric results. UV-Visible spectra (Figure 3) shows indeed increased absorption related in a non-linear fashion to the nominal concentration of titanium butoxide employed with respect to the un-modified sample. The typical α-Fe_2_O_3_ absorption features are preserved in the modified samples, showing in all cases the same absorption threshold, indicating no change in the band gap of hematite by introducing Ti(IV).

**Figure 4:**
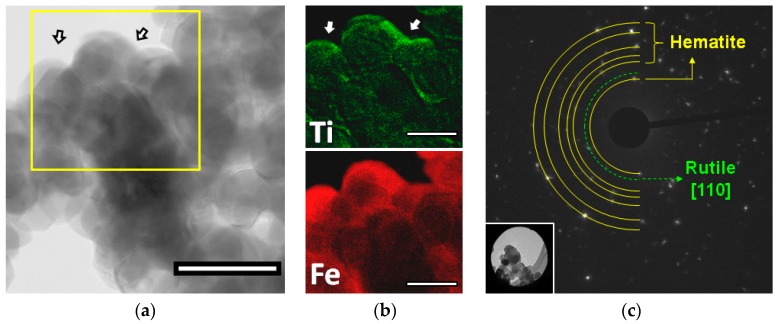
High resolution TEM and SAED imaging. (**a**) TEM micrograph of 10% Ti-modified hematite sample (100 nm scale bar); (**b**) related ESI elemental distribution maps for Ti and Fe (50 nm scale bars); (**c**) Electron diffraction analysis of selected area (in the figure inset below) of 10% Ti-modified hematite sample. The arrows indicate the SAED sampling zones.

In order to investigate the morphological and structural features of modified hematite, the 10% Ti-modified sample was characterized by transmission electron microscopy (TEM). TEM analysis showed the presence of roundish hematite nanoparticles ranging between 25 to 50 nm in diameter confirming the AFM indications about the smoother shape of the nanoparticles in this sample (Figure 4a). High resolution micrographs revealed a homogeneous thin layer onto the surface of all hematite grains (2–7 nm thickness). Elemental ESI maps revealed the presence of titanium (Figure 4b), consistent with the presence of the thin titanium oxide shell highlighted by XPS analysis. Moreover, SAED analysis of the sample (Figure 4c) showed the characteristic diffraction pattern of iron oxide in hematite crystalline phase together with a reflex which can be attributed to the (110) crystal plan of the rutile phase of TiO_2_.

**Figure 5:**
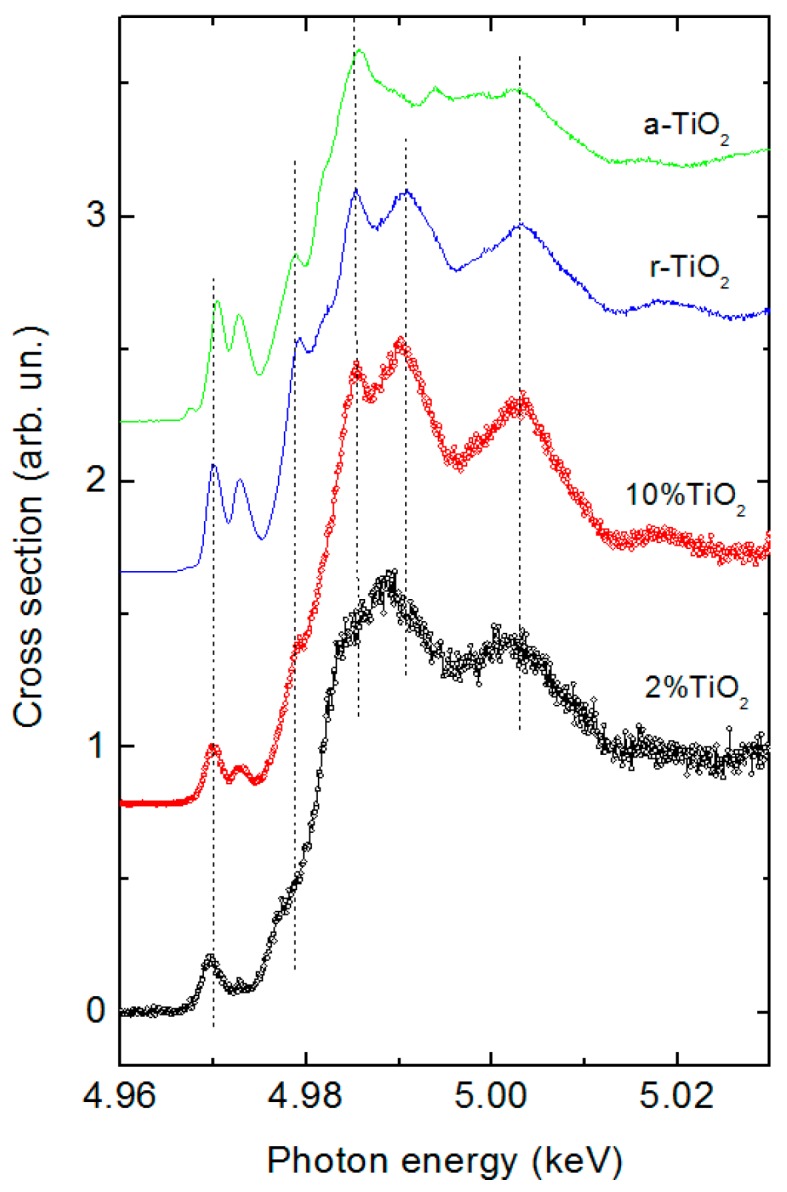
Ti K-edge XANES spectra of 2% a 10% Ti(IV)-modified hematite sample using rutile and anatase (TiO_2_) as references.

Figure 5 compares the Ti K-edge XANES of the 2% and 10% Ti-modified hematite samples with those of anatase and rutile (TiO_2_), taken as reference. For the 10% Ti-modified sample, the energy position of maxima (marked in the figure by the vertical dashed lines) coincides very well with those of rutile, confirming, in this latter case, the presence of a pure TiO_2_ shell on the semiconductor surface, in agreement with SAED analysis. By contrast, samples obtained at a significantly lower concentration, like 2% Ti, do not show a perfect match with neither of the two TiO_2_ reference oxides (anatase and rutile) nor a combination of the two, indicating that the surface composition here bears the probable contribution of a mixed iron titanate one, in the form of FeTiO_3_, as reported by Liu et al. [35]. Such a phase, differently from Fe_2_TiO_3_, would have insulating properties similar to those of TiO_2_, having the valence and conduction band edges respectively more positive and more negative than those of hematite [36,37].

### 2.2. DC Photoelectrochemical Characterization

**Figure 6:**
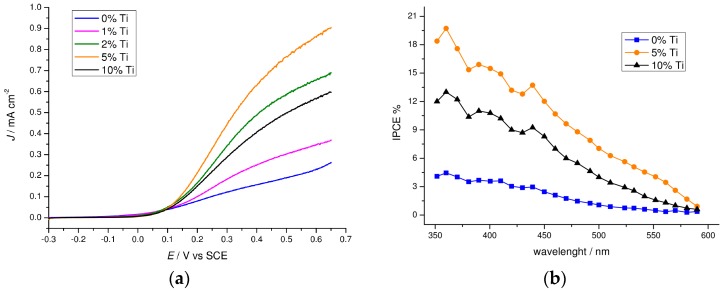
Photoelectrochemical measurements on Ti-modified hematite thin films. (**a**) *J–V* response of un-modified and Ti(IV)-modified hematite samples under AM 1.5G illumination (100 mW·cm^−2^) in NaOH 0.1 M. Nominal titanium concentrations are reported in the legend. (**b**) IPCE spectra of un-modified (0%) and 5% and 10% Ti-modified hematite electrodes recorded at 0.6 V vs. SCE in 0.1 M NaOH (pH 13.3).

**Figure 7:**
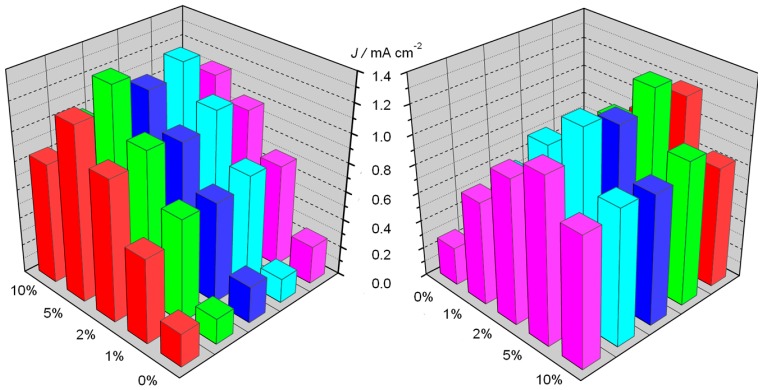
Comparison of the photoactivity of different batches (colored bars) of modified and un-modified hematite electrodes measured under AM 1.5 illumination. The histogram is shown from two different perspectives for clarity. Nominal titanium concentrations are reported in the x-axis.

In order to understand the effect of the Ti(IV) addition during the electrophoretic deposition of the iron oxide nanoparticles, we compared the photocurrent density vs. potential recorded in 0.1 M NaOH (pH 13.3) under AM 1.5G continuous illumination (100 mW·cm^−2^) shining from the FTO side. Interestingly, the introduction of titanium(IV) butoxide results in a significant enhancement of the anodic photocurrent for all the modified hematite samples in comparison with the un-modified hematite (Figure 6a). As the percentage of titanium precursor is increased from 0% to 5%, the photocurrent increases from ca. 0.25 mA·cm^−2^ up to 1.0 mA·cm^−2^ at 0.65 V vs. SCE, decreasing again at larger percentages, probably due to the increased thickness of the TiO_2_ insulating layer. The performance of un-modified hematite is in excellent agreement with previous results obtained using the same deposition technique [8], with a very good reproducibility among different samples (Figure 7). IPCE spectra recorded at 0.65 V vs. SCE confirmed the superiority of the 5% Ti-modified samples, producing a 4-fold improvement of the external quantum yield with respect to simple hematite (Figure 6b), in good agreement with the *J–V* behavior. Given that the absorption at wavelengths <600 nm are for all the samples quantitative (Figure 3), differences in IPCE values are thus ascribable to different charge transfer and charge collection properties, resulting from a lower percentage of photogenerated carriers lost by recombination in the Ti-modified samples. 

**Figure 8:**
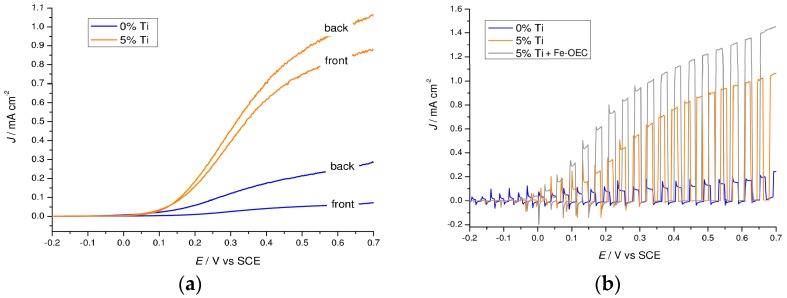
*J–V* curves of modified hematite photoanodes. (**a**) *J–V* comparison between back (FTO side) and front (electrolyte side) illumination for unmodified (blue lines) and 5% Ti-modified (orange lines) hematite electrodes; (**b**) *J–V* curves of unmodified (blue line) and 5% Ti-modified hematite electrode before (orange line) and after (grey line) functionalization with Fe-OEC recorded under shuttered AM 1.5G illumination.

Illumination from the electrolyte side (front side) reduced dramatically the photocurrent in the unmodified hematite, limited to less than 0.1 mA·cm^−2^ at 0.65 V vs. SCE, while this effect is less pronounced in the 5% Ti-modified samples (Figure 8a). Given that the light penetration depth of hematite is reported to be 118 nm at 550 nm, illumination from the electrolyte side generates charge carriers far from the FTO contact resulting in a poor electron collection ability. The presence of titanium oxide significantly alleviates this problem, probably through a variety of effects which include reduction of the grain boundaries, passivation of the surface trap states and surface field effects which cannot be discerned through the analysis of the *J–V* characteristics. After the Surface Ionic Layer Adsorption and Reaction (SILAR) deposition of the iron(III) oxygen evolving catalyst (Fe-OEC) on the 5% Ti-modified photoanode, a 40% increased photocurrent is achieved at 0.65 V vs. SCE, coupled with a ca. 100 mV cathodic shift on the onset potential (Figure 8b). The discontinuous amorphous iron oxide islands onto the hematite surface were demonstrated to store oxidizing equivalents in reactive states exposed to the electrolyte enhancing charge separation [5], consistent with increased photocurrent. Evolution of small oxygen bubbles at the photoanode during constant potential photoelectrolysis (0.15 V vs. SCE) was evident by visual inspection and confirmed with an electrochemical oxygen probe (Figure S3).

### 2.3. Photoelectrochemical Impedance Spectroscopy

**Figure 9:**
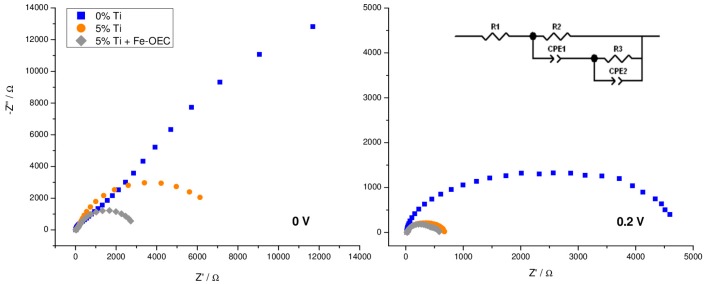
Nyquist plots recorded under AM 1.5G illumination in 0.1 M NaOH (pH 13.3) of un-modified (blue squares) and 5% Ti-modified hematite electrodes before (orange circles) and after functionalization with Fe-OEC (grey diamonds) at two selected potentials vs. SCE. The circuital model used for data fitting is reported as an inset.

EIS was performed by scanning the *J–V* characteristic under illumination, between −0.1 and 0.6 V vs. SCE, where photocurrent generation occurs, in order to highlight the role of titanium oxide surface layer and to address the active elements responsible for charge generation and separation. The best performing Ti-modified electrode (5% Ti-modified sample) was selected for such investigation. Electric equivalents to fit EIS data comprise a nested circuit where two parallel meshes model the charge transfer across the space charge region and the hole transfer through surface trapped intermediates (inset Figure 9). Typical Nyquist plots for the two samples are exemplified in Figure 9, where two different representative potentials along the *J–V* curve were chosen: close to the photocurrent onset (0 V vs. SCE) and in the rising part of the *J–V* curves (0.2 V vs. SCE).

In general, two arcs can be discerned at the lowest sampled potential (from −0.1 to 0.1 V vs. SCE), with the charge transfer arc (low frequency arc on the right side of the plot) dominating over the high frequency one (left side of the plot). At 0.2 V vs. SCE, where photocurrent starts to be noticeable, the low frequency loop decreases for both samples and, in agreement with *J–V* plots, the associated resistance is lower for the 5% Ti-modified sample with respect to the untreated electrode.

**Figure 10:**
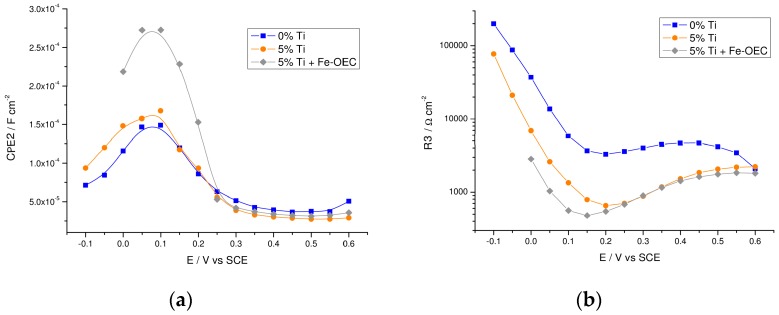
Trapped photohole capacitance and charge transfer resistance extracted from EIS measurements under illumination. (**a**) CPE2 and (**b**) R3 plot of un-modified and 5% Ti-modified hematite electrodes before and after functionalization with Fe-OEC recorded under AM 1.5G illumination in NaOH (0.1 M, pH 13.3).

The inverse of R3, that is the resistance associated to the low frequency arc, obtained by the fitting of EIS data, is in excellent agreement with the differential resistance obtained by the derivative of the *J–V* curves (∂*J*/∂*V*) for both samples (Figure S4), indicating that the low frequency loop, which models the charge transfer across the interface, is mainly responsible for the photocurrent generation and it is thus associated to hole transfer from surface trap states. The trapped photoholes (namely high valent Fe(IV) states acting as potential water oxidation intermediates) capacitance (CPE2) (Figure 10a) show the expected maximum in correspondence of the minimum reached by the charge transfer resistance (R3) (Figure 10b) for both the un-modified and modified samples. The passivation of recombination centers by introducing the titanium oxide based overlayer in the 5% Ti-modified sample only results in a moderate photoholes capacitance increase with respect to the un-modified one, particularly at low potential bias (−0.1–0.1 V vs. SCE). At the same time a ca. 5-fold reduction of the charge transfer resistance occurs (orange curve, Figure 10b), in quantitative agreement with the larger photocurrent observed in the *J–V* curves. Thus, the main feature of the top performing 5% Ti-modified electrode is a substantial reduction in the charge transfer resistance rather than an increase in surface photoholes capacitance. This latter effect, which is after all rather minor, should be the main outcome in the case of radical suppression of charge recombination or increased bulk conductivity, both resulting in a larger density of holes reaching the surface and being trapped there. By contrast, a significant increase in photogenerated hole capacitance (ca. × 2 by considering the capacitance peak) is only observed in the presence of the hole transfer catalyst, consistent with previous observations and with the additional boosting of photocurrent after Fe-OEC treatment. It should be noted that in this latter case the charge transfer resistance also decreases slightly (grey curve in Figure 10b). The introduction of the Ti(IV) oxide overlayer thus improves only slightly surface hole trapping but favors greatly hole transfer from the semiconductor surface into the electrolyte. Such effect could be partly motivated by increased n-type doping associated to surface modification.

**Figure 11:**
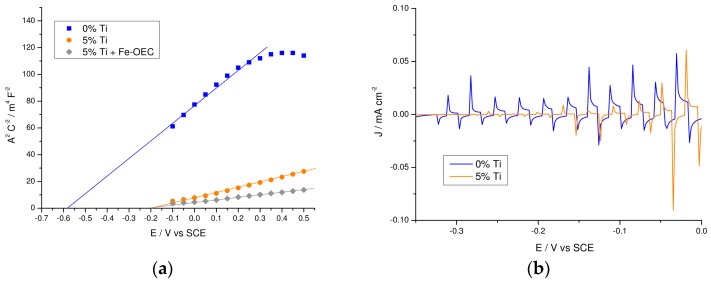
Mott-Schottky plot and photocurrent transients taken in the photocurrent onset potential region. (**a**) Mott-Schottky plot of un-modified and 5% Ti-modified sample before and after functionalization with Fe-OEC recorded at 100 Hz under AM 1.5 G illumination in NaOH 0.1 M (pH 13.3). Fitting lines are reported. (**b**) Magnification of the *J–V* curves recorded under shuttered illumination of un-modified and 5%-Ti modified hematite electrodes.

The high frequency capacitance follows indeed a MS behavior from which the doping density of the films can be extracted (Figure 11a): although the MS intercept should be taken with caution, due to local surface effects on the dielectric constant of hematite, it provides a doping density 3 times higher for the 5% Ti-modified sample with respect to the un-modified hematite (5.04 × 10^18^ and 1.87 × 10^18^ cm^−3^ respectively) resulting in a thinner but deeper depletion layer, where the stronger electric field near the surface facilitates hole extraction. Further, the presence of a stronger surface dipole in the Ti(IV)-modified samples, instrumental in favoring charge separation, is consistent with the positive shift (ca. 400 mV) of the flat band potential in the Ti-modified film, which agrees with the anodically shifted photocurrent transients observed in the −0.35–0 V vs. SCE interval (Figure 11b). Thus EIS data indicate that local electric field effects induced by the presence of pure or mixed titanium oxide surface phases are mainly responsible for enhanced interfacial hole transfer in this type of modified hematite films.

## 3. Materials and Methods

### 3.1. Preparation and Functionalization with Fe-OEC of Un-Modified and Ti(IV)-Modified Hematite Film

Details of the electrophoretic deposition procedure for the preparation of mesoporous hematite films on FTO glass (Fluorine-doped Tin Oxide, TEC 8 Ω·cm^−2^, Hartford Glass, Hartford Glass Co., Hartford City, IN, USA) can be found elsewhere [8]. Briefly, in a Teflon beaker FeCl_3_·6H_2_O (0.55 g, >99%, Sigma Aldrich, St. Louis, MO, USA) was dissolved in a mixed solution of ethanol (20 mL) and water (5 mL) containing sodium acetate (0.8 g, Sigma Aldrich, ≥98%) and kept in a steel autoclave at 180 °C for 12 h. The resulting red powder was washed several times with water and acetone and then suspended in an acetone solution of iodine (Sigma Aldrich, ≥99.8%). Two FTO slides were immersed in the colloidal solution and polarized at 10 V for 35 s in two electrode configuration: the negative electrode, which results in a homogeneous coating of iron oxide nanoparticles, was washed with acetone and then annealed first at 550 °C for 1 h and then at 800 °C for 20 min in air. For the preparation of titanium-modified hematite samples, different amounts of a 10^−2^ M titanium (IV) butoxide (Sigma Aldrich, 97%) solution in ethanol was added to the iron oxide deposition solution just before the application of the 10 V potential. The nominal concentration of the added titanium(IV) added was 0%, 1%, 2%, 5% and 10% with respect to the molar concentration of iron, considering quantitative the conversion of FeCl_3_·6H_2_O in Fe_2_O_3_ nanoparticles during the hydrothermal growth. Different volumes of ethanol were added to the deposition solution to keep constant the acetone/ethanol ratio (49:1). Ti(IV) modified hematite electrodes were functionalized with iron(III) hydrous oxide (generally indicated as (Fe_2_O_3−x_(OH)_x_·*x*(H_2_O), Fe-OEC) [38] by using 10 cycles of SILAR deposition [39]. Each SILAR cycle consisted in the immersion of the electrode for 10 seconds in a 50 mM FeCl_3_·6H_2_O aqueous solution, followed by dipping (10 s) in 0.1 M NaOH (Alfa Aesar, 98%, Thermo Fisher GmbH, Karlsruhe, Germany). After each cycle the electrode was rinsed with abundant distilled water and after the completion of the SILAR cycles it was annealed in air at 200 °C for 20 min.

### 3.2. AFM, XPS, TEM and XANES Characterization

AFM topographical investigation was performed with a Nanoscope III Scanning Probe Microscope (Veeco-Digital Instruments, Bruker, Billerica, MA, USA) equipped with a silicon tip (model RTESP, resonant frequency 300 KHz) and operated in tapping mode with a scan rate of 1 Hz, 512 points resolution and scanned area of 5 μm × 5 μm. 

XPS experiments were conducted using a Φ5600ci X-Ray Photoelectron spectrometer (Perkin Elmer, Waltham, MA, USA) with an AlK_α_ anode X-ray source (1486.6 eV) with a primary beam energy of 14 kV and an electron current of 20 mA. A CHA (Concentric Hemispherical Analyser) was used to collect the output signals. Analysed areas were circles of 0.8 mm in diameter. Survey scan mode acquires spectra stepping every 0.8 eV (pass energy 187.85 eV), while multiplex scan mode requires 0.125 eV steps (pass energy 58.70 eV). A charge neutralizer was used to avoid spectral shift in insulating samples and all spectra were corrected according to charging effect, assigning to C 1s the peak at 284.8 eV binding energy (Figure S1 in the Supplementary Materials).

The TEM sample was prepared scratching with a scalpel the electrode surface and collecting by adherence the nanostructures directly onto a perforated carbon coated copper grid. The specimen was then inserted in the LIBRA200FE column (ZEISS, Oberkochen, Germany) for morphological analysis, selected area diffraction (SAED) and electron spectroscopic imaging (ESI). For ESI elemental maps, data were collected via energy-filtering electrons at the corresponding electron energy loss (EEL) for titanium L and iron L3 edges. The LIBRA200 in column ω-filter spectrometer coupled with iTEM (Olympus-SIS, EMSIS-GmbH, Münster, Germany) software were used applying a three-window methodology.

X-ray absorption spectroscopy experiments were performed on the ID26 beamline at the ESRF, in fluorescence mode in the spectral region near the Ti K-edge (also called XANES: X-ray Absorption Near Edge Structure). Five spherically bent (R = 1 m) Ge crystals in <331> reflection were used to select and focalize the Ti K-β fluorescence (4931.8 eV) on an avalanche photodiode.

### 3.3. AC/DC Photoelectrochemical Characterization

All AC/DC measurements were carried out in a three electrode configuration cell using a platinum and a saturated calomel electrode (SCE) as counter and reference electrode, respectively, and an ABET solar simulator (AM 1.5G, 100 mW·cm^−2^). *J–V* curves were recorded on an Autolab PGSTAT 302/N electrochemical work-station (Eco Chemie, Utrecht, The Netherlands) at a scan speed of 20 mV·s^−1^ by scanning the bias region between the open circuit voltage (under illumination) and 0.7 V vs. SCE in NaOH (0.1 M, pH 13.3). Shuttered *J–V* curves were obtained using a manual shutter (Oriel Instruments-Newport, Irvine, CA, USA). Oxygen evolution measurements in solution under constant potential (0.15 V vs SCE) photoelectrochemical conditions were carried out with a Crison Oxi45 + oxygen probe (Crison Instruments, Alella, Spain). 

IPCE spectra were recorded in NaOH (0.1 M, pH 13.3) under a potential bias of 0.6 V vs. SCE. The photoanodic current was recorded every 10 nm from 350 and 590 nm on a PGSTAT 30 electrochemical workstation. The incident monochromatic irradiance was measured with a calibrated silicon photodiode (Centronic ASD100-7Q, Croydon, UK). IPCE was calculated according to Equation (1):

(1) $$IPCE=1.24\times 10^{-3}(Vm)\frac{J_{\lambda }(\mu Acm^{-2})}{\lambda (nm)P_{\lambda }(Wm^{-2})}$$

where *J*_λ_ is the photocurrent density at the wavelength λ and *P*_λ_ is the incident radiant power. 

Potentiostatic impedance data of photoanodes in the dark and under illumination condition were recorded in NaOH (0.1 M, pH 13.3) from −0.1 to 0.6 V vs. SCE at 50 mV intervals employing a FRA2.v10 frequency response analyzer controlled by Nova 1.10. A 10 mV amplitude sinusoidal perturbation (single sine), whose frequency ranged between 100,000 and 0.05 Hz was adopted. The impedance response was fitted using ZView software (Scribner Associates Incorporated, Southern Pines, NC, USA) with the electric equivalents reported in Figure 10a.

## 4. Conclusions

Nanocrystalline hematite thin films were modified with a Ti(IV) butoxide precursor in solution during the electrophoretic deposition of pre-formed crystalline hematite nanoparticles obtained through a hydrothermal process in aqueous solution. A combination of surface spectroscopic techniques and microscopic imaging revealed that Ti(IV) is deposited onto the surface of the nanoparticles in the form of a nanometric shell of Ti (IV) based materials in the prevailing form of rutile at the highest Ti(IV) concentration (10% nominal Ti/Fe) and in the form of mixture of Ti(IV) oxides, comprising the probable coexistence of rutile and FeTiO_3_, at lower (2%) concentration. In general, all Ti-modified hematite films underwent a significant enhancement in their photoelectrochemical response, with the best results observed at intermediate Ti(IV) butoxide concentration (5%). The comparative EIS investigation of the top performing electrodes provided insights about the role of the titanium oxide surface layer, suggesting that the latter acts partly as a passivating layer for charge recombination, but its main effect is most probably related to the set of surface electric field effects that promote interfacial hole extraction, as indicated by the significant drop in charge transfer resistance. The further treatment of Ti(IV)-modified hematite photoanodes with a hole transfer catalyst based on an amorphous Fe(III) oxide resulted in an additional 40% increase in photoanodic current, reaching values of ca. 1.5 mA/cm^2^ at 0.65 V vs. SCE in 0.1 M NaOH, among the highest recorded with solution processed hematite film in the absence of precious elements.

## Supplementary Materials

Supplementary materials can be accessed at: http://www.mdpi.com/1420-3049/21/7/942/s1.

## Abbreviations

The following abbreviations are used in this manuscript:

B.E.: Binding Energy
EIS: Electrochemical Impedance Spectroscopy
ESI: Electron Spectroscopy Imaging
Fe-OEC: Iron(III)-based Oxygen Evolving Catalyst
FTO: Fluorine Tin-doped Oxide
FWHM: Full Width at Half Maximum
MS: Mott-Schottky
SCLJ: Semiconductor Liquid Junction
SILAR: Successive Ionic Layer Adsorption and Reaction
TEM: Transmission Electron Microscopy
SAED: Selected Area Electron Diffraction

## References

[1] Sivula K., le Formal F., Grätzel M. (2011). Solar Water Splitting: Progress Using Hematite (α-Fe_2_O_3_) Photoelectrodes. ChemSusChem.

[2] Osterloh F.E. (2013). Inorganic nanostructures for photoelectrochemical and photocatalytic water splitting. Chem. Soc. Rev..

[3] Tilley S.D., Cornuz M., Sivula K., Grätzel M. (2010). Light-Induced Water Splitting with Hematite: Improved Nanostructure and Iridium Oxide Catalysis. Angew. Chem. Int. Ed..

[4] Malara F., Minguzzi A., Marelli M., Morandi S., Psaro R., dal Santo V., Naldoni A. (2015). α-Fe_2_O_3_/NiOOH: An Effective Heterostructure for Photoelectrochemical Water Oxidation. ACS Catal..

[5] Dalle Carbonare N., Cristino V., Berardi S., Carli S., Argazzi R., Caramori S., Meda L., Tacca A., Bignozzi C.A. (2014). Hematite Photoanodes Modified with an FeIII Water Oxidation Catalyst. ChemPhysChem.

[6] Sivula K. (2013). Metal Oxide Photoelectrodes for Solar Fuel Production, Surface Traps, and Catalysis. J. Phys. Chem. Lett..

[7] Klahr B., Gimenez S., Fabregat-Santiago F., Bisquert J., Hamann T.W. (2012). Photoelectrochemical and Impedance Spectroscopic Investigation of Water Oxidation with “Co-Pi”-Coated Hematite Electrodes. J. Am. Chem. Soc..

[8] Dalle Carbonare N., Carli S., Argazzi R., Orlandi M., Bazzanella N., Miotello A., Caramori S., Bignozzi C.A. (2015). Improvement of the electron collection efficiency in porous hematite using a thin iron oxide underlayer: towards efficient all-iron based photoelectrodes. Phy. Chem. Chem. Phys..

[9] Liang Y., Enache C.S., van de Krol R. (2008). Photoelectrochemical Characterization of Sprayed α-Fe_2_O_3_ Thin Films: Influence of Si Doping and SnO_2_ Interfacial Layer. Int. J. Photoenergy.

[10] Zandi O., Beardslee J.A., Hamann T. (2014). Substrate Dependent Water Splitting with Ultrathin α-Fe_2_O_3_ Electrodes. J. Phys. Chem. C.

[11] Glasscock J.A., Barnes P.R.F., Plumb I.C., Savvides N. (2007). Enhancement of Photoelectrochemical Hydrogen Production from Hematite Thin Films by the Introduction of Ti and Si. J. Phys. Chem. C.

[12] Hu Y.-S., Kleiman-Shwarsctein A., Forman A.J., Hazen D., Park J.-N., McFarland E.W. (2008). Pt-Doped α-Fe_2_O_3_ Thin Films Active for Photoelectrochemical Water Splitting. Chem. Mater..

[13] Chiam S.Y., Kumar M.H., Bassi P.S., Seng H.L., Barber J., Wong L.H. (2014). Improving the Efficiency of Hematite Nanorods for Photoelectrochemical Water Splitting by Doping with Manganese. ACS Appl. Mater. Interfaces.

[14] Kleiman-Shwarsctein A., Hu Y.-S., Forman A.J., Stucky G.D., McFarland E.W. (2008). Electrodeposition of α-Fe_2_O_3_ Doped with Mo or Cr as Photoanodes for Photocatalytic Water Splitting. J. Phys. Chem. C.

[15] Kumar P., Sharma P., Shrivastav R., Dass S., Satsangi R.V., Khemani D.L., Srivastava M.M., Srivastava S. (2012). Photoelectrochemical Hydrogen Generation using Al Doped Nanostructured Hematite Thin Films. Chemistry of Phytopotentials: Health, Energy and Environmental Perspectives.

[16] Liu J., Liang C., Xu G., Tian Z., Shao G., Zhang L. (2013). Ge-doped hematite nanosheets with tunable doping level, structure and improved photoelectrochemical performance. Nano Energy.

[17] Kim T.-H., Kim H.S., Hwang I.-C., Yoon K.B. (2014). Effect of metal doping, doped structure, and annealing under argon on the properties of 30 nm thick ultrathin hematite photoanodes. Phy. Chem. Chem. Phys..

[18] Munetoshi S., Hiroyasu Y., Hitoshi T. (2012). Enhanced Photocurrent in Rh-Substituted α-Fe_2_O_3_ Thin Films Grown by Pulsed Laser Deposition. Appl. Phy. Express.

[19] Shen S., Zhou J., Dong C.-L., Hu Y., Tseng E.N., Guo P., Guo L., Mao S.S. (2014). Surface Engineered Doping of Hematite Nanorod Arrays for Improved Photoelectrochemical Water Splitting. Sci. Rep..

[20] Wang G., Ling Y., Wheeler D.A., George K.E.N., Horsley K., Heske C., Zhang J.Z., Li Y. (2011). Facile Synthesis of Highly Photoactive α-Fe_2_O_3_-Based Films for Water Oxidation. Nano Lett..

[21] Atabaev T.S., Ajmal M., Hong N.H., Kim H.-K., Hwang Y.-H. (2014). Ti-doped hematite thin films for efficient water splitting. Appli. Phys. A.

[22] Fu Z., Jiang T., Liu Z., Wang D., Wang L., Xie T. (2014). Highly photoactive Ti-doped α-Fe_2_O_3_ nanorod arrays photoanode prepared by a hydrothermal method for photoelectrochemical water splitting. Electrochim. Acta.

[23] Hahn N.T., Mullins C.B. (2010). Photoelectrochemical Performance of Nanostructured Ti- and Sn-Doped α-Fe_2_O_3_ Photoanodes. Chem. Mater..

[24] Rioult M., Magnan H., Stanescu D., Barbier A. (2014). Single Crystalline Hematite Films for Solar Water Splitting: Ti-Doping and Thickness Effects. J. Phys. Chem. C.

[25] Miao C., Shi T., Xu G., Ji S., Ye C. (2013). Photocurrent Enhancement for Ti-Doped Fe_2_O_3_ Thin Film Photoanodes by an In Situ Solid-State Reaction Method. ACS Appl. Mater. Interfaces.

[26] Deng J., Zhong J., Pu A., Zhang D., Li M., Sun X., Lee S.-T. (2012). Ti-doped hematite nanostructures for solar water splitting with high efficiency. J. Appl. Phys..

[27] Zandi O., Klahr B.M., Hamann T.W. (2013). Highly photoactive Ti-doped α-Fe_2_O_3_ thin film electrodes: Resurrection of the dead layer. Energ. Environ. Sci..

[28] Franking R., Li L., Lukowski M.A., Meng F., Tan Y., Hamers R.J., Jin S. (2013). Facile post-growth doping of nanostructured hematite photoanodes for enhanced photoelectrochemical water oxidation. Energy Environ. Sci..

[29] Ahmed M.G., Kretschmer I.E., Kandiel T.A., Ahmed A.Y., Rashwan F.A., Bahnemann D.W. (2015). A Facile Surface Passivation of Hematite Photoanodes with TiO_2_ Overlayers for Efficient Solar Water Splitting. ACS Appl. Mater. Interfaces.

[30] Monllor-Satoca D., Bartsch M., Fabrega C., Genc A., Reinhard S., Andreu T., Arbiol J., Niederberger M., Morante J.R. (2015). What do you do, titanium? Insight into the role of titanium oxide as a water oxidation promoter in hematite-based photoanodes. Energ. Environ. Sci..

[31] Hu S., Shaner M.R., Beardslee J.A., Lichterman M., Brunschwig B.S., Lewis N.S. (2014). Amorphous TiO_2_ coatings stabilize Si, GaAs, and GaP photoanodes for efficient water oxidation. Science.

[32] Zong X., Thaweesak S., Xu H., Xing Z., Zou J., Lu G., Wang L. (2013). A Scalable Colloidal Approach to Prepare Hematite Films for Efficient Solar Water Splitting. Phys. Chem. Chem. Phys..

[33] Fujii T., de Groot F.M.F., Sawatzky G.A., Voogt F.C., Hibma T., Okada K. (1999). In situ XPS analysis of various iron oxide films grown by NO_2_-assisted molecular-beam epitaxy. Phys. Rev. B.

[34] Augustynski J., Alexander B.D., Solarska R., Bignozzi C.A. (2011). Metal Oxide Photoanodes for Water Splitting. Photocatalysis.

[35] Liu F., Asakura K., Xie P., Wang J., He H. (2013). An XAFS study on the specific microstructure of active species in iron titanate catalyst for NH_3_-SCR of NO_x_. Catal. Today.

[36] Bassi P.S., Gurudayal, Wong L.H., Barber J. (2014). Iron based photoanodes for solar fuel production. Phys. Chem. Chem. Phys..

[37] Kim Y.J, Gao B., Han S.Y., Jung M.H., Chakraborty A.K., Ko T., Lee C., Lee W.I. (2009). Heterojunction of FeTiO_3_ Nanodisc and TiO_2_ Nanoparticle for a Novel Visible Light Photocatalyst. J. Phys. Chem. C.

[38] Cristino V., Berardi S., Caramori S., Argazzi R., Carli S., Meda L., Tacca A., Bignozzi C.A. (2013). Efficient solar water oxidation using photovoltaic devices functionalized with earth-abundant oxygen evolving catalysts. Phys. Chem. Chem. Phys..

[39] Kim H., Seol M., Lee J., Yong K. (2011). Highly Efficient Photoelectrochemical Hydrogen Generation Using Hierarchical ZnO/WOx Nanowires Cosensitized with CdSe/CdS. J. Phys. Chem. C.

