# Supplementary material for: Photoelectrochemical Behavior of Electrophoretically Deposited Hematite Thin Films Modified with Ti(IV)
Source: Molecules. 2016 Jul 20;21(7):942. doi: 10.3390/molecules21070942 (PMC6273019; doi:10.3390/molecules21070942)
Supplement: Supplementary file 1 [file molecules-21-00942-s001.pdf]

Supplementary Materials: Photoelectrochemical Behavior of Electrophoretically Deposited Hematite Thin Films Modified with Ti(IV)

Nicola Dalle Carbonare, Boaretto Rita, Stefano Caramori, Roberto Argazzi, Maurizio Dal Colle, Luca Pasquini, Renzo Bertoncello, Marcello Marelli, Claudio Evangelisti and Carlo Alberto Bignozzi

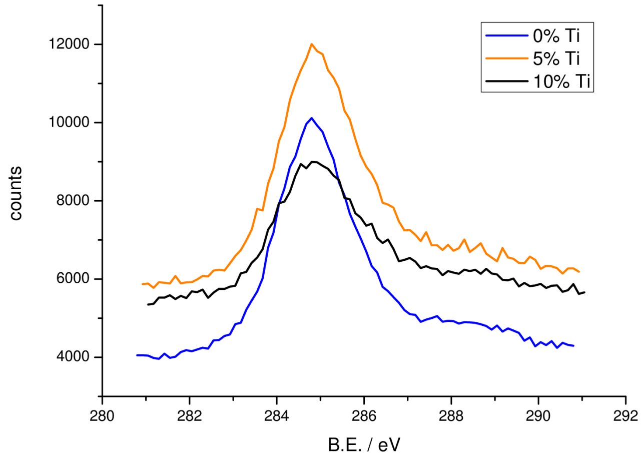

Figure S1. C 1s core level XPS spectra for un-modified and Ti-modified hematite films (5% and 10%).

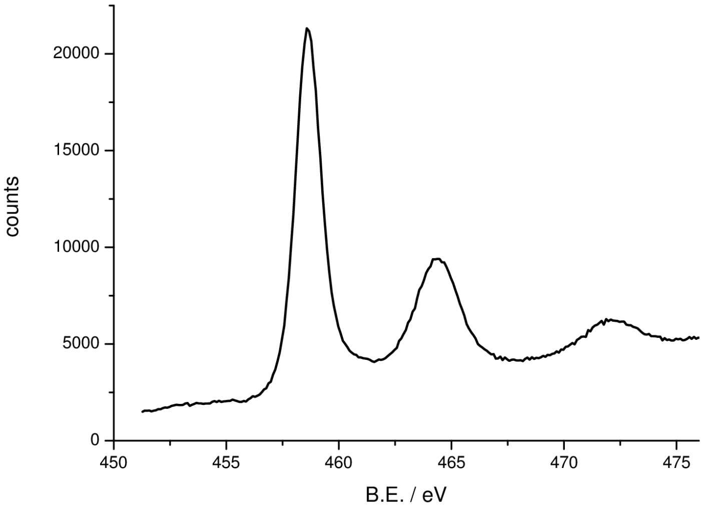

Figure S2. Ti 2p core level XPS spectra of TiO_2.

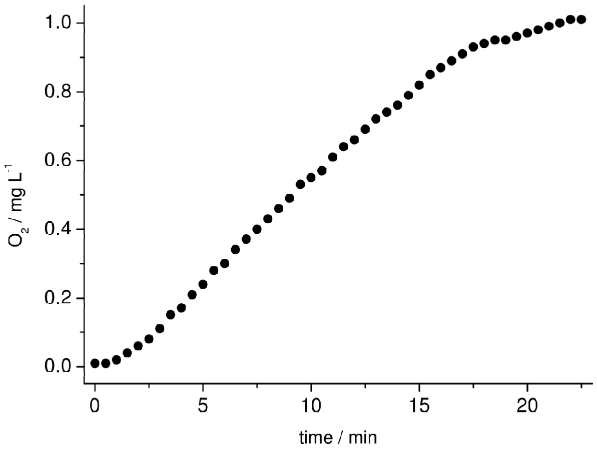

Figure S3. Typical oxygen evolution at a Ti(IV) modified hematite photoanode functionalized with Fe-OEC biased at 0.15 V vs. SCE under AM 1.5 G illumination in 0.1 M NaOH.

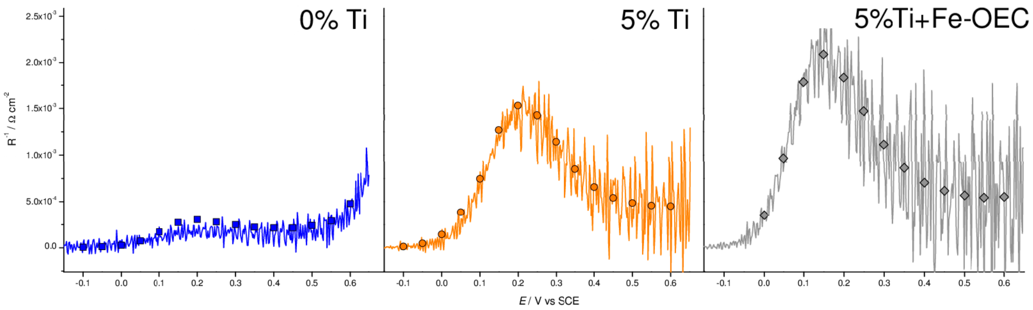

Figure S4. Derivatives of the *J-V* curve (∂*J*/∂*V*, solid lines) plot together with the inverse of R3 values (symbols) obtained from the EIS data fitting of the low frequency loop for the un-modified and 5% Ti-modified before and after functionalization with Fe-OEC.
